# Characterization of age-related modifications of upper limb motor control strategies in a new dynamic environment

**DOI:** 10.1186/1743-0003-5-31

**Published:** 2008-11-19

**Authors:** Benedetta Cesqui, Giovanna Macrì, Paolo Dario, Silvestro Micera

**Affiliations:** 1 Lucca Institute for Advanced Studies, IMT, Italy; 2 Advanced Robotics Technology and Systems Lab, Scuola Superiore Sant'Anna, Pisa, Italy; 3 Institute for Automation, Swiss Federal Institute of Technology, Zurich, Switzerland

## Abstract

**Background:**

In the past, several research groups have shown that when a velocity dependent force field is applied during upper limb movements subjects are able to deal with this external perturbation after some training. This adaptation is achieved by creating a new internal model which is included in the normal unperturbed motor commands to achieve good performance. The efficiency of this motor control mechanism can be compromised by pathological disorders or by muscular-skeletal modifications such as the ones due to the natural aging process. In this respect, the present study aimed at identifying the age-related modifications of upper limb motor control strategies during adaptation and de-adaptation processes in velocity dependent force fields.

**Methods:**

Eight young and eight elderly healthy subjects were included in the experiment. Subjects were instructed to perform pointing movements in the horizontal plane both in a null field and in a velocity dependent force field. The evolution of smoothness and hand path were used to characterize the performance of the subjects. Furthermore, the ability of modulating the interactive torque has been used as a paradigm to explain the observed discoordinated patterns during the adaptation process.

**Results:**

The evolution of the kinematics during the experiments highlights important behavioural differences between the two groups during the adaptation and de-adaptation processes. In young subjects the improvement of movement smoothness was in accordance with the expected learning trend related to the consolidation of the internal model. On the contrary, elders did not show a coherent learning process. The kinetic analysis pointed out the presence of different strategies for the compensation of the external perturbation: older people required an increased involvement of the shoulder with a different modulation of joint torque components during the evolution of the experiments.

**Conclusion:**

The results obtained with the present study seem to confirm the presence of different adaptation mechanisms in young and senior subjects. The strategy adopted by young subjects was to first minimize hand path errors with a secondary process that is consistent with the optimization of the effort. Elderly subjects instead, seemed to shift the importance of the two processes involved in the control loop slowing the mechanism optimizing kinematic performance and enabling more the dynamic adaptation mechanism.

## Background

Beside its apparent simplicity, moving the upper limb toward a target requires the coordination and the regulation of many biomechanical variables, which rule joint arm motion, such as interaction torques (IT), and inertial resistance [1]. There is now a general consent on the idea that when human subjects are asked to move in new or perturbed environments a representation – called "internal model" (IM) – of the relationship between the arm state of motion and the external perturbation is generated and/or updated by the central nervous system (CNS) in order to achieve the desired trajectory of the arm [2]. The IM is learnt with practice and appears to be a fundamental part of the voluntary motor control (MC) strategies [3,4]. In this context, several studies analyzed the mechanisms influencing its efficacy; dedicated experiments have been carried out asking subjects to perform "center-out" bidimensional pointing movements either in visually or mechanically distorted environments, or with different loads [5-8]. The knowledge gained during these experiments can be useful to help people to restore motor functions when it is compromised for example for neurological disorders (e.g., stroke, Parkinson's disease) or for traumatic brain injuries.

The same approach can also be used to understand the modifications of MC strategies due to the natural aging process. However, age-related modifications in motor control strategies are not easy to be detected throughout a simple observation of motor behavior because aging does not affect a specific part or function of motor control system. Conversely, it modifies the whole body system in terms of: morphological degradation of neural tissues, decreased number of Type II (fast twitch) muscle fibers and their associated motor neurons; reduced efficiency of the sensory system, which limits the performance during complex motor tasks [9]; disturbances in temporal organization of motor synergies and postural reflexes; decreased maximum rate of sequential repetitive movements [10]; and impaired performance in tasks requiring complex programming and transformations [11]. Most noticeable consequences of these changes are an increased delay in reacting to environmental stimuli and in making voluntary movements. Rapid movements are usually more slowly initiated, controlled, and concluded, coordination is also disrupted [12].

This situation poses the question of whether and how elderly subjects develop alternative strategies in the coordination of upper limb movements to overcome their physical modifications and to adapt to different environmental conditions. Past works dealing with this problem evaluated elders performance while reacting to visual distorted environments or different hand speed. It has been observed that simultaneous shoulder and elbow control during aiming movements is less efficient in subjects of advanced age [13]. In fact, the co-activation of antagonist muscles when both joints were involved determined a difficulty in the regulation of the interaction torque (IT), which affects movement coordination. In particular, this behavior is more evident at higher movements frequencies when IT substantially increases. In addition, other studies [14,15] observed that old adults tend to decrease the production of muscle force to overcome a perturbation. They also showed the ability to compensate this limit by using a sophisticated joint control strategy which relies more on shoulder movements and less on the elbow.

Furthermore, researchers dealing with adaptation to a modified visual environment [16] showed that older adults can learn new motor skills and that there are two distinct processes: acquisition (learning of a new process) and transfer (ability to use what has been learned to new task demands); aging affects motor acquisition but not saving based on past experience. In this respect, Bock and Girgenrath [8], asserted that this reduced adaptive ability was partly due to the decay of basic response speed and decision making, and partly to age-dependent phenomena not related to cognitive causes. Up to now, to our knowledge, no one studied elders adaptation to a velocity dependent force field. Contrary to visual perturbation which causes a modification of the perceived kinematics of movements, changing the mechanical environment interacting with the subject hand requires an adaptation of the IM to the new dynamics [17].

In this work, upper limb kinetic and kinematic behaviors were analyzed in young and elderly subjects performing pointing movements while interacting with a velocity dependent force field environment. In particular, the effects of adaptation and de-adaptation were analyzed to characterize differences in motor control strategies developed by the two groups to overcome the external perturbation. In this respect, the evolution of hand trajectories, the regulation of the ITs and the modulation of joint torques were used to quantify the capability and the efficiency of recalibrating the IM. Our results seem to show that aging affects the relationship between kinematic and dynamic optimizations during the adaptation, shifting the priority between the two processes.

## Methods

### Subjects

Eight healthy right handed elderly subjects (Group 1, 72 ± 5 years old), and eight right handed young subjects (Group 2, 24 ± 4 years old) were recruited for the present study. All volunteers received a brief explanation of the experimental protocol before starting and signed an informed consent in accordance with the policies about trials with human subjects.

### Procedure

Each participant seated on a chair and gripped the handle of a planar manipulandum, the Inmotion2 Robot (Interactive Motion Technologies Inc., Boston, MA, USA), used to guide and perturb movements during the experiment. Trunk movements were prevented by means of a belt, while the elbow was supported in the horizontal plane by an anatomical orthosis. Subjects were instructed to move from the centre of the workspace forward and backward to reach eight different targets positioned every 45° on the perimeter of a circle with a 14 cm diameter. Subjects performed pointing exercises both in null force field (NF) and in a velocity-dependent force field (VF):

(1) $$\begin{array}{ccc} \text{F}=\text{K*v,} & \text{with} & K=\left[\begin{array}{cc} 0 & \lambda \\ -\lambda & 0 \end{array}\right] \end{array}$$

where forces were always orthogonal to hand velocity, forming a clockwise curl field (λ = 20 N s/m, v = hand speed). Such experimental paradigm has been used in several studies on motor control adaptation in altered force fields environments [4,18,19].

Each subject involved in the study performed a total of 832 movements corresponding to 52 turns, divided into the following experimental session:

#### Session 1: Null field environment

exercise 1: Familiarization (2 turns to take confidence with the robotic device)

exercise 2: Learning unperturbed dynamics (20 turns in NF to learn how to move in this condition)

#### Session 2: Velocity dependent force field environment

exercise 3: Early learning (4 turns in VF field)

exercise 4: Adaptation (20 turns in VF field)

#### Session 3: Null field environment

exercise 5: De-Adaptation (4 turns in NF field)

exercise 6: Final Washout (2 turns in NF field).

Two further elderly subjects (group 1.2, 70 and 81 years old) executed the same protocol doubling the number of trials in exercise 5 of session 3 (de-adaptation phase). This approach was used to verify whether difference between the two groups at the end of the experiment could be related to fatigue or other physical factors.

Participants were instructed to perform movements in the most ecological way. During the experiment an audio feedback was given when they went too slow or too fast so that movement speed remained always between 0.15 m/s and 0.4 m/s. The purpose of this approach was to make them execute the exercise in the most natural way, in order to observe the real strategy adopted during the adaptation, but trying to obtain comparable performance inside each group. Visual feedback of target position while performing the exercises was given by a computer screen located in front of the subject. No explicit instructions regarding the hand path were given. Movements were recorded with the use of an Optotrak 3D optoelectronic camera system (Optotrak 3020, Northern Digital, Waterloo, Ontario Canada), and collected considering each trial as the displacement from the center to the goal point and back at 200 Hz sampling rate. The infrared diodes were positioned in four anatomical landmarks: trunk (sternum), shoulder (acromio), elbow, and wrist (considered as the end point).

### Data analysis

Data were low-pass filtered (fifth order Butterworth filter, zero-phase distortion; MATLAB "butter" and "filtfilt" functions). Hand position was differentiated to compute speed, acceleration and Jerk profiles. Movement onset and offset were detected when the end-point velocity exceeded 5% of the peak velocity value. Shoulder and elbow joint angular displacements, velocities and accelerations were also determined. Positive direction of motion was assigned to flexion and negative to extension. Both kinetic and kinematic analyses were carried out by looking in a specific way at the different movement directions. In fact, other research groups [20] have shown that the anisotropy and orientation of inertia ellipse of the upper limb determines movements characterized by higher inertia in left diagonal direction, and by higher accelerations in right diagonal direction. To evaluate the efficiency of movements a normalized length path parameter was calculated with the following Equation [21]:

(2) *LL *= (Σ*dR*)/*L*_*t*_

where dR is the distance between two points of the subject's path and L_t _is the theoretical path length, represented by the distance of the two extreme points of the stroke. Higher values of LL correspond to hand trajectories affected by larger errors.

The smoothness parameter N.Jerk was also computed using the metric proposed by Teulings and coworkers which consists of the time- integrated squared jerk opportunely normalized [22]:

(3) $$N.Jerk=\sqrt{\left(\frac{1}{2}\int dt\;j^{2}\times duration^{5}/length^{2}\right)}$$

where j is the Jerk, that is the change of the acceleration per time, and it is calculated as the third time derivative of position. This parameter has the advantage to be dimensionless and usable to compare movements with different characteristics (i.e., duration, size). Reduced coordination results in multiple acceleration peaks at the base of an increase of the jerk levels, hence, the lower the parameter, the smoother the motion.

For each group, and for each movement direction the mean value and standard deviation of the movement smoothness have been computed within all the exercise sessions; in exercise 2 and 4 only the values of the last 5 trials were used in order to evaluate the values achieved after the consolidation of the learning process.

A simplified model of the arm based on the Newton-Euler [23] recursive algorithm, was used to compute the torque acting at the shoulder and the elbow. Anthropometric measure of limb were took into account in the computation of the joint torques: segmental masses, location of mass centre and moments of inertia were estimated from he weight and the height of the subjects in accordance with Winter [24]. Torques estimated at each joint with this model were grouped according to the approach proposed by Dounskaia et al. [14]: 1) net torque (NT), proportional to the angular acceleration at the joint; 2) interaction torque (IT), that depends on motion at both joint and on the nature of the force field in which subjects moved; 3) muscle torque (MUSC) which considers the muscle activity and the viscoelastic properties of the entire arm. In particular, the Equations for torque computation at the joints are:

(4) *MUSE*_*E *_= *NT*_*E *_- *IT*_*E *_- *IT*_*field*_

(5) *MUSE*_*S *_= *NT*_*S *_- *IT*_*S *_- *MUSC*_*E*_

where _S _and _E _apexes represent the shoulder and elbow joints; IT_field _= 0 when the field is turned off. To investigate the role of the MUSC, IT and IT_field _components in motion production, a sign analysis was computed in accordance with previous works by Dounskaia and co workers [14,25]. Shortly, the torque sign analysis determines the percentage of time when the analyzed torque (MUSC or IT) has the same sign of the NT torque, i.e., it gives a positive contribution to movement acceleration and it is responsible for it. To provide information about the magnitude of the contribution of MUSC to the NET, the difference between positive and negative peaks of the MUSC torque was computed for both joints hence after called MT magnitude. The evolution of all these parameters (LL, N.Jerk, elbow and shoulder torques sign, and magnitude values) was monitored throughout the experiment in order to observe the macroscopic effects of different motor control strategies adopted by each person and group. Performance achieved by each subject at the end of exercise 2 were considered as a reference, i.e. subjects after being trained for a prolonged time in an unperturbed environment achieved the most ecological motion. Indeed, differences in kinematic and kinetic trends between exercise 2 and all the other phases were considered as a consequence of the presence of the external perturbation; their evolution during adaptation and de-adaptation was, then, used to quantify efficiency of the motor strategies adopted.

### Statistical analysis

T-test on joint excursions was computed to evaluate differences between elders and young. For each of the eight directions an overall ANOVA 2 × 6 (group × exercise) was computed both for hand speed peak value, the torque sign indexes. Fisher test on exercise 2 and 4 (the ones relative to the NF and VF characterize by a sufficient higher number of samples) was computed to see whether the angular coefficient of the linear regression between velocity and the number of turns was significantly different from 0; this test was performed with the twofold objective of: 1) verifying whether hand speed varied throughout the consolidation exercises; 2) for exercise 4, quantifying the relative changes in force field perturbation. Post-hoc tests (Bonferroni correction) were conducted to perform pair wise comparison both on hand speed peak value and MT magnitude.

## Results

Elbow and shoulder mean excursion values and the SD for each direction are shown in table 1. The t-test (p = 0.94) did not reveal a significant group effect. Shoulder excursions were not so wide due to the short displacement required by the experiment. During the experiments, hand speed was in the range 0.22 – 0.38 m/s for young subjects, and in the range 0.15 – 0.3 m/s for old subjects. The characteristics of hand motion are listed below: 1) young subjects were always faster than elders (see table 2); 2) in accordance with literature [14,20], subjects went faster moving toward right directions; 2) young subjects moved faster when the field was applied (exercise 4 – consolidation of VF), than when it was turned off (exercise 2- consolidation of NF); on the contrary in VF condition elderly subjects (a part in NE direction), maintained the same speed values observed in NF case and in some cases they even moved slowly (see table 2); 4) there was a significant variation of young subjects hand speed both within the learning sessions, i.e. exercises 2 and 4 (Fisher test: p < 0.01 in all direction both in exercises 2 and 4). In particular, subjects tended to go slightly faster at subsequent turns: as a consequence in exercise 4 they increased the intensity of the perturbation force applied by the robot of 24.1% with respect to mean value measured in exercise 2. Elderly population instead maintained the same hand velocity throughout all exercise 2, and poorly increased its value during exercise 4 only in 4 of the 8 directions: compared to young group they showed lower coefficients of the linear regression between the peak of speed and the exercise turn (Fisher test: p > 0.05 in all direction on exercise 2 and in 4 direction of exercise 4).

**Table 1:** Mean values and standard deviation of elbow and shoulder joints excursions for each movement direction.

Movement direction		Mean value
N	Elbow	-22,3 ± 4,53
	Shoulder	10,85 ± 3,5
NE	Elbow	-21,72 ± 4,43
	Shoulder	-4,5 ± 1,8
E	Elbow	-2,33 ± 2,09
	Shoulder	-9,22 ± 3,06
SE	Elbow	16,87 ± 3,14
	Shoulder	-10,14 ± 3,97
S	Elbow	24,97 ± 2,23
	Shoulder	-1,21 ± 1,37
SW	Elbow	14,65 ± 5,93
	Shoulder	9,32 ± 3,11
W	Elbow	-6,07 ± 2,82
	Shoulder	12,57 ±
NW	Elbow	-19,6 ± 3,11
	Shoulder	8,54 ± 3,58

**Table 2:** Mean value and SD of the hand effecter for each age group and each direction.

	Ex	N	NE	E	SE	S	SW	W	NW
**Young subjects Hand Speed**	2	0,28 (± 0,04)	0,29 (± 0,04)	0,28 (± 0,04)	0,27 (± 0,04)	0,27 (± 0,05)	0,27 (± 0,04)	0,27 (± 0,04)	0,29 (± 0,04)
	
	3	0,28 (± 0,04)	0,32 (± 0,05)	0,28 (± 0,05)	0,25 (± 0,04)	0,29 (± 0,04)	0,29 (± 0,03)	0,28 (± 0,04)	0,26 (± 0,04)
	
	4	0,32 (± 0,04)^+^	0,34(± 0,04)^+^	0,31 (± 0,04)^+^	0,28 (± 0,04)^+^	0,31 (± 0,03)^+^	0,31(± 0,04)^+^	0,31 (± 0,04)^+^	0,3 (± 0,04)^+^
	
	5	0,27 (± 0,04)^+^	0,26(± 0,04)^-^	0,31 (± 0,08)^-^	0,27 (± 0,03)	0,27 (± 0,03)	0,26 (± 0,03)	0,29 (± 0,03)	0,3 (± 0,03)
	
	6	0,3 (± 0,05)	0,3 (± 0,06)	0,32 (± 0,04)^+^	0,31(± 0,03)^+^	0,3(± 0,05)*	0,3(± 0,05)*	0,32 (± 0,04)^+^	0,33(± 0,04)^+^

**Elderly subjects Hand Speed**	2	0,23 (± 0,04)	0,22 (± 0,05)	0,23 (± 0,04)	0,22 (± 0,04)	0,22 (± 0,04)	0,23 (± 0,04)	0,23 (± 0,04)	0,23 (± 0,04)
	
	3	0,20(± 0,04)^+^	0,22 (± 0,03)	0,20(± 0,03)^+^	0,17(± 0,02)^+^	0,19 (± 0,02)^+^	0,20 (± 0,02)^+^	0,19 (± 0,02)^+^	0,17(± 0,02)^+^
	
	4	0,21 (± 0,04)^+^	0,25 (± 0,04)^+^	0,22 (± 0,04)	0,19 (± 0,03)^+^	0,19 (± 0,05)^+^	0,22 (± 0,03)	0,22 (± 0,04)	0,2 (± 0,02)^+^
	
	5	0,2 (± 0,04)^-^	0,19 (± 0,03)^+^	0,21 (± 0,04)	0,2 (± 0,02)*	0,19 (± 0,03)^+^	0,2 (± 0,03)^-^	0,23 (± 0,04)	0,22 (± 0,04)
	
	6	0,21 (± 0,04)	0,2 (± 0,03)	0,22 (± 0,05)	0,21 (± 0,04)	0,2 (± 0,05)	0,2 (± 0,05)	0,23 (± 0,03)	0,22 (± 0,04)

A Bonferroni post hoc test was made to see when there was a statistical difference with exercise 2. Results showed that young subjects go faster when the field was applied and except for 2 directions, they maintained this attitude in the final washout. Elderly instead in many cases even reduced the speed of their movements when the field was applied; no statistical differences were found between the second and the sixth exercise.

*p < 0.05/4, ^-^p < 0.01/4,+p < 0.001/4

The t-Test made on the length line parameter showed that there were not significant differences on the entity of errors committed by elderly and young subjects in each of the experiment sessions (p = 0.27).

### Smoothness analysis

**Figure 1:**
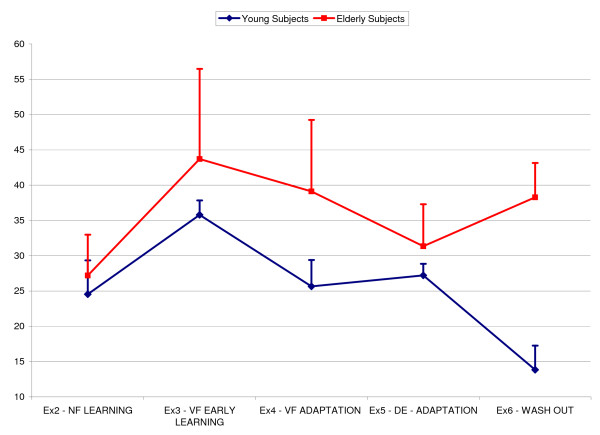
**Evolution of the of the smoothness parameters N.Jerk throughout the experiment in one of the eight direction**. Blue line = young group; red line = elderly group.

In Figure 1 the comparison between the evolution of the smoothness throughout the experiments for the two groups it is shown. The t-Test revealed a significant group effects, i.e. elders were less smooth than young subjects and exercise session effect on the smoothness parameter.

In addiction the two age groups evolved differently throughout the entire experiment see figure 1. In fact, in the case of young subjects, N.Jerk varied in accordance with the expected learning trend. Once trained in the NF condition (exercise 2), subjects achieved a smoother and faster performance characterized by lower N.Jerk values; turning on the VF field, at the beginning of the adaptation (exercise 3) their end point motion was dramatically perturbed and N.Jerk increased significantly. The prolonged exposition to VF environment condition (exercise 4) let improve again the quality of motion almost up to the level observed in the second session. The de-adaptation process and the final washout (exercises 5–6) were then characterized by a decrease of the N.Jerk parameter: young subjects after few trials were able to recover the kinematics and thanked to the prolonged training became always more proficient moving faster and smoother with respect to what observed in the exercise 2.

**Figure 2:**
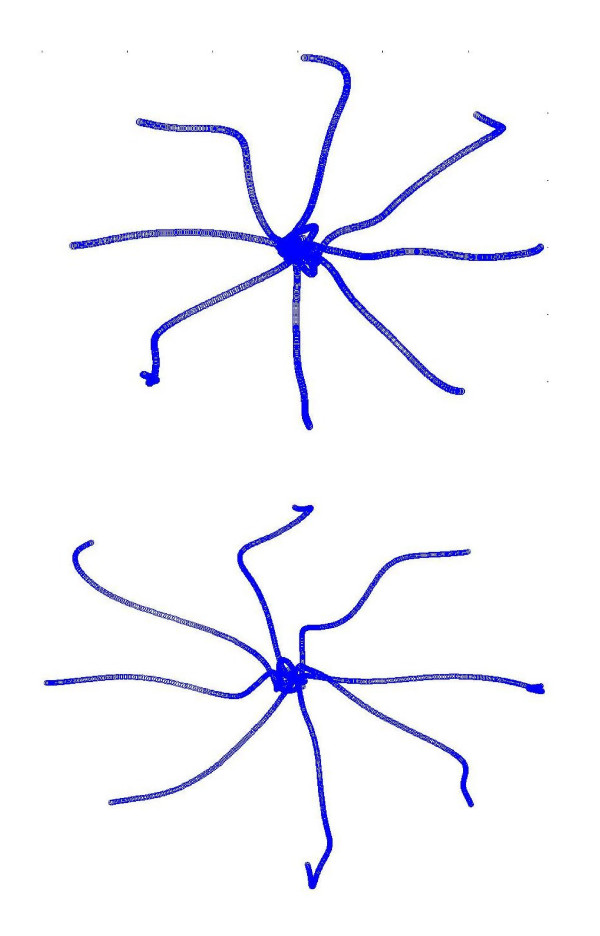
**Hand path trajectories traced by elderly subjects**. a) soon after the field application (exercise 3). b) when the field was turned off (exercise 5).

The analysis of elderly end point trajectories during the early adaptation and de-adaptation phases showed the presence of after-effects, demonstrating that aging does not affect the capability to adapt (figure 2). Nevertheless differences were observed throughout the experiment and specially during the de-adaptation process: N.Jerk in the sixth exercise was higher than in the second one, and passing from the fifth to the sixth exercises it did not vary and in many cases it increased (see figure 1).

**Figure 3:**
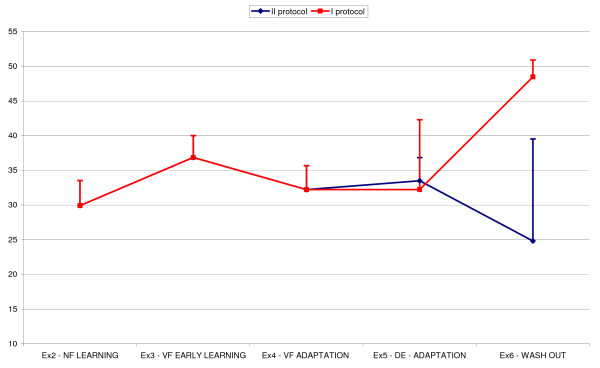
**Comparison between the two different experimental protocols**. Red line is relative to the first adopted experiment protocol. Blue line shows the behaviour in the second version of the experiment protocol, when subjects prolonged de adaptation phase in exercise 5.

In order to verify whether elders did not achieve the same performance as young subjects only because of fatigue, two more elderly subjects where included in the experiment. They were subjected to the same protocol but with a double number of trials in exercise 5. In figure 3 the N.Jerk trend throughout the exercises is represented in one of the eight directions. The blue line represents N.Jerk profile with the new extended experiment protocol, while the red line was traced grouping the data as specified in the previous experiment, with a less number of movements. When subjects performed a higher number of trials (blue line) the evolution of their movement smoothness behaved in the same way observed for young group in figure 1; at the end of the relearning phase movement kinematics was completely restored and the final washout (exercise 6), showed a lower N.Jerk value with respect to the beginning of the training session (exercise 2). If instead subjects performed only 4 turns instead of 8 (red line), at the end of the re-adaptation phase they were not able to completely recover.

### Torque Sign Analysis

**Figure 4:**
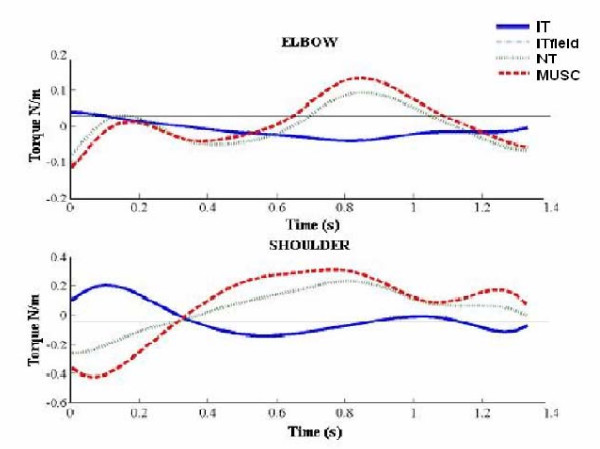
**Individual torque profiles at the shoulder and at the elbow of relative to motion toward right direction**. Positive values correspond to flexion torques and negative values to extension. Upper side: NF condition; Bottom side: VF field condition.

The modulation of IT, MUSC and NET torques in NF and VF conditions was evaluated. Figure 4 shows shoulder and elbow torques profiles, both in NF and VF condition, of one young subject moving in one direction. For both groups, the shoulder was guided mainly by MUSC_S_: when moving in NF, MUSC_S _and NET_S _torque had the same direction and time peaking, while IT_S _was in opposite direction: this means that MUSC_S _compensated for IT_S _and provided for NT_S_. At the elbow in NF condition there were three possible cases: 1) MUSC _E _coincided in sign with elbow net torque (NT _E_) and suppress the opposite effects of IT _E_; 2) IT _E _coincided in sign with NT _E _and MUSC _E_, elbow motion depends also on the shoulder motion; 3) IT _E _coincided in sign with NET _E _and MUSC _E _had the opposite sign, the elbow was guided mainly by the shoulder.

**Figure 5:**
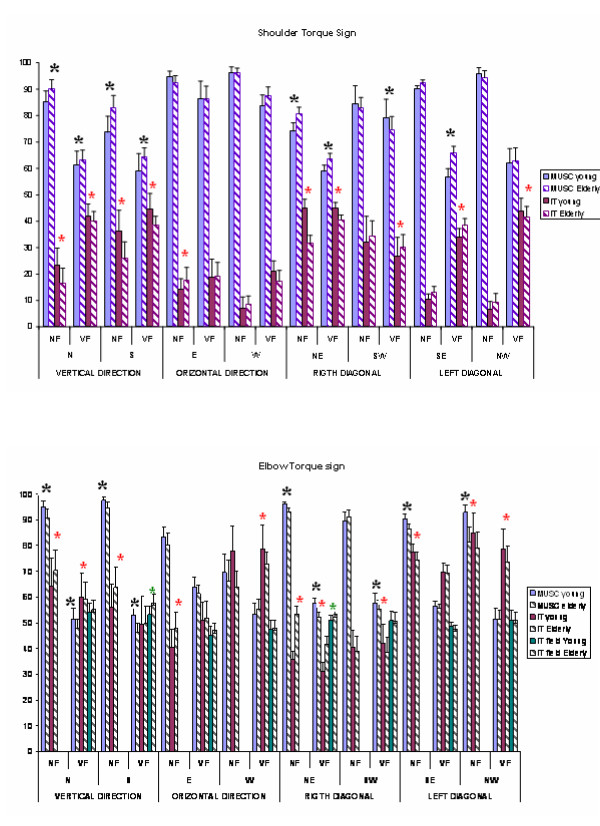
**Torque sign analysis**. Mean percentage of movement duration for the elbow and shoulder during which MUSC or IT coincided in sign with NT. The asterisks indicate when the differences between young and elders are significant.

When the force field was applied the IT_field _component at the elbow quantifies the entity of the contribution of the field to arm motion. The higher its sign index the more influenced and perturbed the motion. For everyone of the 8 directions the NF and VF field conditions, figure 5 shows the mean portions of movement duration for the elbow and the shoulder in which the MUSC, IT, and IT_field_, coincide in sign with NF in both the environment conditions.

### NF Condition

In comparison with the results presented in [14,26], shoulder joint excursions in this study were smaller and the elbow played a more active rule. Actually, small shoulder amplitudes resulted in lower IT_S _at the elbow that demanded for MUSC _E _to suppress IT _E_. Elderly MUSC_S _index was significantly higher or equal to the one presented by young subjects while MUSC _E _index was always smaller see figure 5. Contrary to the other directions, wen shoulder excursions were larger, as in the horizontal and left diagonal directions, MUSC _E _shared the control with IT_S_, as revealed by the higher IT _E _sign index.

The ANOVA test 2 × 6 (group × exercise) revealed for MUSC _E _index a significant difference between the two groups except for the E, W and SW directions which presented a wider shoulder excursion. Elders IT _E_indexes were significantly bigger with respect to young subjects in all the directions except for NW, W, and SW. These results showed that older people relied more on shoulder to control elbow motion. When moving toward **r**ight diagonal direction the elbow acted as leading joint (see table 1): MUSC_S _and MUSC _E _index values were respectively smaller and higher with respect to other directions (figure 5). A similar behavior was observed also in the S direction.

### VF Condition

At both joints it was possible to observe a loss of synchronism between MUSC and NT torques comoponents; in fact in addiction to motion production, MUSC had to compensate for the external perturbation, so that its sign index presented lower values with respect to NF condition. In quite all the directions, passing from NF to VF condition, MUSC_S _sign index significantly decreased (p < 0.01), while instead, a part for the right direction, IT_S _increased, (see figure 5). In general, when the shoulder presented a consistent excursion, IT_field _at the elbow was mainly contrasted by shoulder contribution so that the IT _E _sign index was higher then MUSC _E _index (see figure 5, horizontal and left diagonal directions). Vertical directions (N and S) presented a IT_field _sign index> MUSC _E _index: here, contrary to others directions, motion was affected more by the field; similar considerations can be inferred in the case of movements toward NW direction (IT_field _sign index = MUSC _E_).

Finally, in the directions characterized by smaller shoulder excursions and wide elbow motion (NE and S), IT_field _of elderly population was significantly higher with respect to the one presented by young group, (p = 0.011 in NE direction, p < 0.001 in south direction); no significant differences were found in all other conditions. These results suggested that elders contrasted better the field when the shoulder could contribute more to the motion.

### MT Analysis

**Figure 6:**
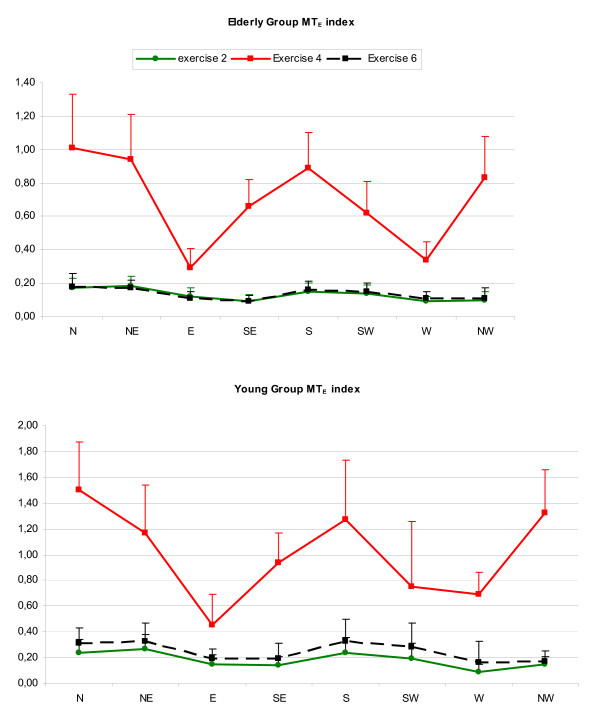
**MT _E _values for elderly and young groups in adaptation and de- adaptation phases**. Bottom side: after the removal of the field (exercise 6) young subjects continued to move with a MUSC _E _torque higher than necessary: differences between exercise 2 and 6 are significant in all direction except W; upper side: elders soon restored the more economic solution in terms of effort.

The magnitude of the MUSC torques was monitored throughout the experiment. The value presented in exercise 2 was considered as reference, as previously explained. The presence of the force field made MUSC_S _and MUSC _E _increase both for elderly and young subjects (see figure 4). The main differences between the two groups were found in the modulation of elbow torques at the end of the relearning phase. The comparison between MT _E _values of both young and elderly participants showed that, while the former, a part for W direction, maintained a higher value of MUSC _E _in the final washout (MT _E _index in exercise 6 > MT _E _index in exercise 2, see figure 6) the latter tended to restore the more economic solution in terms of effort after the removal of the perturbation In this respect, as confirmed by the statistical analysis no significant differences were found in the MT _E _values between exercises 2 and 6.

## Discussion

### Elderly subjects need more trials to restore the correct kinematics

In this study subjects moved their arms in eight directions and in different mechanical conditions. The analysis of the length line parameter, quantifying the entity of the errors in hand path with respect to the ideal trajectories, showed that there were no significant differences between the two groups. That is because the main discontinuities and differences were found more in hand speed. This result justified the need to monitor subject performance through parameter based on velocity and jerk metrics, as a measure the quality of movements. The analysis carried out by using the N.Jerk parameter suggested that, even if the adaptation to a new dynamic environment was not compromised by aging, elderly subjects ability to restore the correct movement kinematics both in the learning (from NF to VF condition) and in the re-learning (from VF to NF condition) phases is altered. Notwithstanding a minor intensity of the perturbation (elders moved always slower with respect to young subjects), they were not able to completely recover the kinematic of motion.

In particular, elderly subjects in the fifth and sixth exercises did not improve their performance as expected. In fact, they did not varied N.Jerk values in the sixth exercise compared to the second one, and in several cases they even increased it. Performance were improved only when the number of trials of the relearning phase was increased. Therefore, results coming from the second protocol analysis confirm that the behavior observed in aged population at the end of the experiment was not due to fatigue and seem to suggest instead that more training is needed to optimized the re-learning process.

### There are differences in torque modulation between young and elderly subjects

#### NF condition

Previous studies evidenced that elders adapt joint control in a specific way for each direction, depending on the specific role of IT in movement production along different directions and asserted that changes in joints control introduced by elders facilitated active control decreasing the demand of MUSC torque [26]. This was achieved at the elbow by exploiting the mechanical interaction between upper and lower arms. Indeed, the IT_S _caused by the shoulder motion can give a bigger contribution with respect to MUSC _E _in the production of elbow joint motion. The torque sign analysis in NF condition confirmed this attitude, because elderly IT _E _and MUSC_S _sign indexes were always bigger in elders with respect to young in almost all the directions.

#### VF condition

Elderly subjects were less affected by the field perturbation (IT_field_sign index in elderly < IT_field _sign index in young) when they could rely on shoulder movements. This is the case of motion toward E, W, SW, NW directions where the active role of the shoulder significantly contributed to elbow movement providing the torque IT_S _to fully compensate the field.

Ketcham at al [26], observing age related modifications in joint control while drawing circles and lines at different speeds, suggested that young and elderly subjects presented two different strategies. Young adults increased the MUSC _E _magnitude, also relatively close in time to IT, and add it to IT_S_. Together the two torques would both increase magnitude and early onset of the NT _E _peaks which easily allows to compensate for IT _E _. Elderly subjects instead were reluctant to increase the magnitude of MUSC _E _torque more than necessary, but activated it early in time to compensate IT, and preventing the excessive increases in NT _E _magnitude. The strategy adopted at high cycling frequency seems to be the same adopted to contrast the force field of our experiments where the elbow often played an active role in movement execution, and field compensation. When the perturbation was applied young subjects produced a MUSC _E _higher than necessary so that in addiction to compensate for the field their speed was larger, although this implies a larger perturbing force. On the contrary older people tried to spend less effort optimizing the interaction between shoulder and elbow: in this context, the contribution of IT_S _has been exploited to decrease the demand for a larger elbow MUSC _E_. The increased MUSC_S _contribution to motion, confirmed by the torque sign analysis, was a consequence of this strategy adopted to compensate the field. The presented theory could explain also what happened in the sixth exercise in terms of MUSC _E _magnitude and N.Jerk parameter. Our results suggest that young subjects after a prolonged training in the perturbing field learned to move producing a MUSC _E _torque larger than necessary and maintained this attitude also in the relearning phase, so that movements were characterized by larger acceleration and velocity, probably at the base of a lower N.Jerk parameter.

Elderly subjects instead soon after the exposition to the external perturbation tended to restore the original torque magnitude in order to spend less effort. When the field was turned off their performance remained characterized by the presence of sub-movements resulting in higher N.Jerk values, which were even more accentuated because the number of trials was, probably, not enough to restore the correct kinematics.

#### Different motor control strategies

The present analysis showed that aging causes delays in the reorganization of MC which resulted in changes in torques modulation, compensation of IT and difficulty in restoring the correct kinematic path. One explanation of this behavior could be related to a general slowing factor at the base of lower feedback signals; having more difficulty in distinguish signals from noise in sensory and perceptual information, older adults can be expected to be slower on tasks that require an efficient feedback to decrease errors from imprecise monitoring and adjustment of movements [27].

Moreover, the observed behaviors could be related also to the relative importance that different mechanisms have in the learning process. Scheidt et al [28] observed that during the adaptation to a velocity dependent force field, when kinematic errors (after-effects) were allowed to occur after the removal of the field, the recovery was faster; instead, when the kinematic errors were prevented subjects persisted in generating large forces that were unnecessary to perform an accurate reach. The magnitude of these forces decreased slowly over time, at a much slower rate than when subjects were allowed to make kinematic errors, hence, two learning states referring to two different control loop seem to act simultaneously. De-adaptation after learning a dynamic force field consists of a rapidly switching between these motor control behaviors. Davidson and Wolpert [29] observed that after learning a dynamic force field, subjects took longer to de-adapt when the forces were turned off than to adapt to a scaled down version of the field. This suggested that de-adaptation reflects a capacity to scale down the relative contribution of existing control modules to the motor output.

Results obtained in this study are consistent with the idea that young subjects tried to minimize hand path errors during movement, while providing evidence for a slower, secondary process that is consistent with the optimization of the efforts or other kinetic criteria. Elderly subjects could shift the importance of the two processes involved in the control loop slowing the mechanism optimizing kinematic performance and enabling more the dynamic adaptation mechanism. Similar results were observed in a recent study by Emken et al [30], who showed that during adaptation to a novel dynamic in walking, motor system coordinates two different processes minimizing a cost function that includes muscle activation and kinematic errors. This theory could explain why elderly performance did not improve, but it does not address the fact that in many cases their performance get worse in the sixth exercise. When subjects are asked to skip from a task to another one, our brain should suppress the activation of no longer relevant goals or information and prevent proponent candidates for response from controlling thought and action. Hasher and Zacks [31] suggested that aging seems to modify this inhibitory mechanism in such a way that made the CNS be influenced by dominant response tendency. In this respect, the presence of a response to stimuli that are no longer relevant to current goals could have compromised in our experiment older subjects ability to quickly recover from the field in the relearning phase; this interpretation is of course speculative and needs to be proved by dedicated experimental trials.

## Conclusion

The results of this work show that aging does not significantly affect the learning process but it strongly influences the way a new IM is learnt. In particular, they seem to imply the presence of competition at retrieval processes affecting CNS behavior. Seniors can adapt and de-adapt to new environment conditions; however our results are consistent with the idea that elderly subjects switch the importance of concurrent mechanisms that contribute to skill learning, in order to reduce their effort. Further experiments will be carried out to understand whether the reduced inhibition process observed in older subjects could be explained by a mechanism that increases the activation of the prime response or by a process that affect the activation of interfering information that allows the brain to switch between different IM models.

## Abbreviations

CNS: Central Nervous System; IM: Internal Model; MC: Motor Control; MUSC* : Muscle torque; NT*: Net torque component; IT*: Interaction torque component; MT*: Magnitude torque index; NF: Null Field environmental dynamic condition; VF: Velocity dependent Force field environmental dynamic condition;N: North direction; NE: North East direction; E: E direction; SE:South East direction; S: South direction; SW: South West direction; W: West direction; NW: North West direction; * _S _and _E _apexes: shoulder and elbow values

## Competing interests

The authors have not competing interests as defined by the BioMed Central Publishing Group, or other interests that may influence results and discussion reported in this study.

## Authors' contributions

BC conceived and designed the study, carried out the experiments and the data analysis and drafted the manuscript; GM participated in the design of the study and carried out the experiment; PD participated in the coordination of the study;

SM conceived of the study, and participated in its design and coordination.

All authors read and approved the final manuscript.
